# Adaptive immunity and atherosclerosis: aging at its crossroads

**DOI:** 10.3389/fimmu.2024.1350471

**Published:** 2024-04-15

**Authors:** Roy P. M. Snijckers, Amanda C. Foks

**Affiliations:** Division of BioTherapeutics, Leiden Academic Centre for Drug Research, Leiden University, Leiden, Netherlands

**Keywords:** adaptive immunity, atherosclerosis, aging, immunosenescence, inflammaging

## Abstract

Adaptive immunity plays a profound role in atherosclerosis pathogenesis by regulating antigen-specific responses, inflammatory signaling and antibody production. However, as we age, our immune system undergoes a gradual functional decline, a phenomenon termed “immunosenescence”. This decline is characterized by a reduction in proliferative naïve B- and T cells, decreased B- and T cell receptor repertoire and a pro-inflammatory senescence associated secretory profile. Furthermore, aging affects germinal center responses and deteriorates secondary lymphoid organ function and structure, leading to impaired T-B cell dynamics and increased autoantibody production. In this review, we will dissect the impact of aging on adaptive immunity and the role played by age-associated B- and T cells in atherosclerosis pathogenesis, emphasizing the need for interventions that target age-related immune dysfunction to reduce cardiovascular disease risk.

## 1 Introduction

Aging leads to an overall impairment of physiological systems due to a gradual decline of cells in terms of function and viability (1). Aging of the immune system, termed “immunosenescence”, induces a skewing of hematopoietic stem cells (HSCs) towards myelopoiesis, diminishing the lymphoid cell pool (2). Alongside a reduction in proliferative naïve T- and B cells and relative increase in central memory cells, the B- and T cell receptor repertoire is reduced upon aging, resulting in decreased antigen responses (3, 4). Furthermore, the adaptation of a senescence associated secretory profile (SASP) by immune cells further contributes to a chronic low-level inflammation termed “inflammaging” (5). Collectively, immunosenescence impairs the response to physiological stress in the aging population while increasing susceptibility to infections and inflammatory disease (6, 7). Aging has additionally emerged as one of the most prominent risk factors for cardiovascular disease (CVD). Specifically, in 2019, the annual rate of cardiovascular-related death was increased by more than 80-fold when comparing age groups of 15-49 years to 70 years and older, whilst being responsible for more than half of all deaths in the latter (8). Atherosclerosis, a lipid-driven chronic inflammatory disease, lies at the heart of CVD pathogenesis (9). Whereas initiation of atherosclerotic plaques often occurs upon damage to the endothelium and subsequent infiltration of lipids into the vessel wall, its progression is marked by the infiltration of immune components leading to chronic inflammation of the plaque (10). Over time, the formation of necrotic debris, plaque destabilization and eventual rupture drive potentially fatal acute cardiovascular events such as a myocardial infarction or stroke (11). In light of the gradual functional decline of the aging immune system, it comes as no surprise that the incidence of acute cardiovascular events also greatly increases with age, even though atherosclerotic vascular changes already start occurring during early adolescence (12, 13). Understanding the interplay between aging, adaptive immunity, and atherosclerosis has significant implications for understanding and addressing age-related cardiovascular complications. Preclinical research has paved the way for this understanding, providing mechanistic insights into the role of adaptive immunity in atherosclerosis pathogenesis. Nevertheless, the majority of preclinical research into atherosclerosis is performed using young adult mice models of 3-6 months of age, equivalent to 20-30 years in humans (14). This subsequently poses translational problems to the actual atherosclerotic cardiovascular disease patient population seeking treatment at advanced age. The characterization of atherosclerotic development in available aging mouse models has shown promising potential in bridging this gap of knowledge. It has been shown previously that aging promotes atherosclerosis development in low density lipoprotein receptor (Ldlr) deficient mice as well as in Apolipoprotein E (ApoE) deficient mice (15–17). Similarly, C57BL/6 mice treated with PCSK9-AAV to induce hypercholesterolemia at advanced age, show increased atherosclerosis development compared to their young counterparts (18). Particularly in chow-diet fed aged Ldlr^-/-^ mice, plaques gradually developed in the context of mild hypercholesterolemia, and show human-like plaque features such as fibrosis and calcification (15). In addition, the immune system ages in these mice, displaying both immunosenescence and inflammaging, providing a clinically relevant setting for elucidating the impact of the aging immune system on atherosclerosis progression (19). With a rising life expectancy and a rapidly increasing aging population, enhancing our understanding of age-associated immunity has developed into a major public health priority.

In this review, we provide a comprehensive overview on the current state of knowledge regarding the changing role of adaptive immunity in atherosclerotic plaque development upon aging. First, the scene will be set through a discussion on the importance of adaptive immunity in atherosclerosis pathogenesis and the hallmarks of immunosenescence. Subsequently, atherosclerosis specific T- and B cell dynamics will be dissected in the context of the aging cell and deteriorating lymphoid tissues. Finally, the potential use of immunoaging as a biomarker or therapeutic target to combat atherosclerotic CVD will be discussed.

## 2 Adaptive immunity in atherosclerosis

The hallmark feature of atherosclerotic plaque initiation is considered to be the accumulation of low density lipoproteins (LDL) in the tunica intima. This can occur due to a "leaky" endothelial cell layer of the vessel wall in response to damage, for example at sites of shear stress (20). Modification of LDL, primarily oxidation (oxLDL), promotes the recruitment and infiltration of monocytes into the vessel wall, and the subsequent accumulation of cholesterol-enriched foam cells that contribute to plaque growth and necrotic core formation (21, 22). Adaptive immune responses, carried out by T- and B cells, play a crucial role in atherosclerosis progression (23, 24).

### 2.1 T cell immunity in atherosclerosis

Distinct subsets of T cells, both effector memory and regulatory T cells (Tregs), influence plaque development and stability. Notably, interferon gamma (IFNγ) secreting T helper (Th) 1 cells are the most common T cells found in atherosclerotic plaques. Th1 cells are considered pro-atherogenic, partially due to their role in stimulating macrophage polarization towards pro-inflammatory M1 effector cells (25). Conversely, interleukin (IL) 10 and transforming growth factor-β secreting Tregs exhibit clear anti-atherogenic functions, such as the inhibition of pro-atherogenic effector T cells as well as the stimulation of efferocytosis by macrophages (26, 27). This atheroprotective role of Tregs may however evolve into maladaptive immunity as Tregs autoreactive to Apolipoprotein B 100 (ApoB100), the main protein in LDL particles, can lose the expression of CD25 and forkhead box P3 (FoxP3) in hyperlipidemic ApoE^-/-^ mice. In atherosclerosis, these so-called exTregs have been shown to exhibit a pro-inflammatory Th1/Th17 phenotype with cytotoxic properties (28, 29). A fraction of exTregs can additionally convert to T follicular helper (Tfh) cells during atherogenesis dependent on the accumulation of intracellular cholesterol (30). The role of Th2 and Th17 cells can be either pro- or anti-atherogenic, dependent on the cytokines they secrete (31–33). Similarly, cytotoxic CD8^+^ T cells display pro-atherogenic effects through IFNγ secretion and stimulating monopoiesis but can additionally exert an atheroprotective function by regulating Tfh cell mediated germinal center (GC) B cell responses as well as contributing to plaque stability (34–36). Advances in single cell technology further support the importance of adaptive immunity in atherosclerosis and revealed T-cells to be the most abundant leukocyte present in human carotid atherosclerotic plaques, outnumbering myeloid populations (37). Additionally, T cell receptor (TCR) sequencing has exposed plaque specific clonal expansion of CD4^+^ effector T cells with transcriptome profiles indicative of recent antigen-mediated T cell activation, thus suggesting an autoimmune component in atherosclerosis pathology (38).

The contribution of T cells to pathogenic immunity is not only restricted to the atherosclerotic plaque. Rather, their involvement in the activation of B cells at secondary lymphoid organs (SLOs), such as the lymph nodes or the spleen, plays a significant role (22, 39). Here, naïve T cells are primed by follicular dendritic cells (FDCs) in T cell zones, leading to their differentiation towards pro-atherogenic Tfh cells (40–42). C-X-C Motif Chemokine Ligand (CXCL) 13 dependent localization of Tfh cells towards the T-B cell border allows for their direct interaction with B cells (43–45). Symbiotic IL-21/IL21R and CD40/CD40L signaling further solidifies commitment to the Tfh lineage and drives FO B cell differentiation towards GC B cells (42, 46). Through this interaction, Tfh cells drive the humoral response by aiding in antibody affinity maturation through stimulation of somatic hypermutation and class switch recombination (42). Upon differentiation into plasmablasts and plasma cells, B cells influence atherosclerosis progression via the production of auto-antibodies targeting oxLDL and necrotic debris (47). Tfh cells are counteracted by follicular regulatory T helper cells (Tfr) which express both Tfh markers such as Bcl-6 and CXCR5 for migration on the CXCL13 gradient as well as the Treg marker FoxP3 (48). Initially, Tfr also express the Treg marker CD25, but down regulate its expression upon maturation and migration towards the germinal center, favoring the expression of Tfh related genes (49). The adoptive transfer of Tfr cells in ApoE^-/-^ mice has resulted in a decrease of atherosclerotic plaques alongside a decrease in infiltrating macrophages as well as a decrease in the amount of splenic Tfh cells whilst stimulating the expansion of atheroprotective regulatory B cells (50).

### 2.2 B cell immunity in atherosclerosis

Research concerning the atherogenicity of B cells has highlighted the existence of both pro-atherogenic and atheroprotective B cell subsets. B2 cells are the most prominent subset present within the B cell population of the circulation and SLOs. B2 cells are subdivided into follicular B cells (FO B cells) and marginal zone B cells (MZ B cells) which either stimulate or inhibit plaque progression, depending on the cell type. FO B cells mainly contribute to the germinal center response through their interaction with Tfh cells, differentiating into GC B cells to produce high affinity immunoglobulin (Ig) G autoantibodies against (neo) self-antigens (51). Previously, a 50% decrease in FO B cells was achieved through stimulation of the B- and T-lymphocyte attenuator (BTLA) pathway, which significantly diminished atherosclerotic plaque size in Ldlr^-/-^ mice (52). Additionally, a lack of FO B cells induced by CD23-specific Blimp-1 or Pax5 deletion leads to a reduction in IgG and atherosclerotic lesion size, while IgG from atherosclerotic mice, compared to IgG derived from non-atherosclerotic mice, increased plaque size when injected into Ldlr^-/-^ mice lacking endogenous IgG (53, 54). IgG autoantibodies targeting oxidation specific epitopes (OSEs) such as heat shock protein (HSP) 60/65 overexpressed by stressed endothelial cells as well as products of oxLDL breakdown such as malondialdehyde, 4-hydroxynenal and phosphocholine-containing oxidized phospholipids have furthermore been shown to increase due to hyperlipidemia (54, 55). As the polar opposite to FO B cells, MZ B cells inhibit Tfh cells, suppressing their atherogenic functionality while producing atheroprotective IgM in a T-cell dependent manner (56, 57). B1 cells, composed of B1a and B1b cells, similarly stand among the anti-atherogenic B cell subsets by producing substantial amounts of germline encoded IgM natural antibodies targeting OSEs. Through OSE binding, these natural antibodies facilitate efficient phagocytosis of oxLDL and apoptotic cells in non-immunogenic ways (58). Lastly, IL-10^+^ regulatory B cells have been shown to inversely correlate with plaque severity (59). Nevertheless, whether the atheroprotective role of regulatory B cells is IL-10 dependent is unclear as B cell–specific deficiency in IL-10 has not been shown to affect lesion size or composition in Ldlr^-/-^ mice (60).

Summarizing, adaptive immunity significantly impacts atherosclerosis pathogenesis, with T- and B cells playing pivotal roles. T cell subsets such as Th1 cells are pro-atherogenic, while Tregs exhibit anti-atherogenic functions but can become maladaptive in hyperlipidemic conditions. Additionally, T cells facilitate humoral responses through interacting with B cells in SLOs, aiding in the production and affinity maturation of IgG auto-antibodies against (neo) self-antigens. B cell subsets have dichotomous effects on plaque progression, with FO B cells promoting it and Bregs as well as OSE-specific IgM producing MZ B cells and B1 cells offering atheroprotection. Understanding the nuances of adaptive immunity in atherosclerosis is crucial for developing targeted therapeutic interventions.

## 3 Immunosenescence

As we age, the components of our immune system undergo significant changes in terms of viability and function, potentially impacting the role they play in CVD. Several immunological changes can be included among the cardinal features of immunosenescence such as an increase in central memory cells alongside a decrease in peripheral naïve T- and B cells, increased autoimmune responses by cells of the adaptive immune system and a reduction in the capacity for antigen stimulated immune responses, severely impacting the susceptibility for infectious disease and age-associated inflammatory diseases in the elderly population (3–7). Additionally, immunosenescence induces an increase in chronic systemic inflammation caused by changes in the secretory profile of senescent immune cells, i.e. the SASP (5). To understand these aging-associated changes, it is however of vital importance to first discuss the most commonly established mechanisms underlying immunosenescence, before diving deeper into the effects of aging on T- and B cells in atherosclerosis.

### 3.1 The “first hit” paradigm

The “first hit” paradigm, as postulated by Franceschi et al. in 1997, encapsulates the concept of chronic exposure to stressors leading to a gradual increase in pro-inflammatory status over time, thereby augmenting the risk of age-related diseases (61). These stressors are represented by physical, chemical and biological agents which constantly assault organisms. Among these stressors, exogenous factors, such as x-rays, ultraviolet light, and a variety of chemicals; as well as endogenous reactive metabolites consistently impede on cellular viability (62, 63). Protection is granted through the DNA damage response system, antioxidants and the immune system while the individual variety in genetic disposition conveys variable susceptibility to specific stressors, aging and disease. This chronic exposure to stressors can be partially attributed to natural chronological aging, representing the passage of time. As such, some features of immunosenescence are inherent to chronological aging, including both thymic involution and replicative immunosenescence which will be discussed in detail in the next sections (64, 65). Nevertheless, biological aging, encompassing the multifaceted interplay of various agents influencing the aging process, is profoundly affected by lifestyle factors such as diet, exercise, and smoking, as well as various comorbidities which can induce premature biological aging in terms of stress-induced senescence, cholesterol-induced changes in hematopoiesis and excessive DNA damage (66, 67). Similarly, chronic infections, for example with cytomegalovirus (CMV), can lead to immunosenescence, increasing senescent CD4^+^ CD28^-^ T cells, as well as increasing CVD risk (68–70). An increased risk for complications related to ischemic CVD has similarly been observed to present itself in a cohort of older individuals following infections with SARS-CoV-2 (71). Chronic infections with HIV or human gamma herpes viruses, such as Epstein-Barr virus, furthermore lead to an increase in age-associated immune subsets, such as age-associated B cells (ABC), which could contribute to inflammatory diseases (72).

### 3.2 Aging-induced changes in hematopoiesis

Immunosenescence is a main contributor to the “first hit” by driving inflammation. One of the most prominent features of immunosenescence becomes apparent through aging-induced changes in the hematopoietic output of primary lymphoid tissues, namely the bone marrow and thymus. The bone marrow has a critical function in producing common lymphoid progenitor cells (CLPs) during hematopoiesis and serves as the primary site for B cell development (73). However, with age, the quality of HSCs drops in terms of regenerative capacity, impacting self-renewal. This in turn leads to a reduction in the output of naïve T- and B cells from the bone marrow (74).

**Figure 1:**
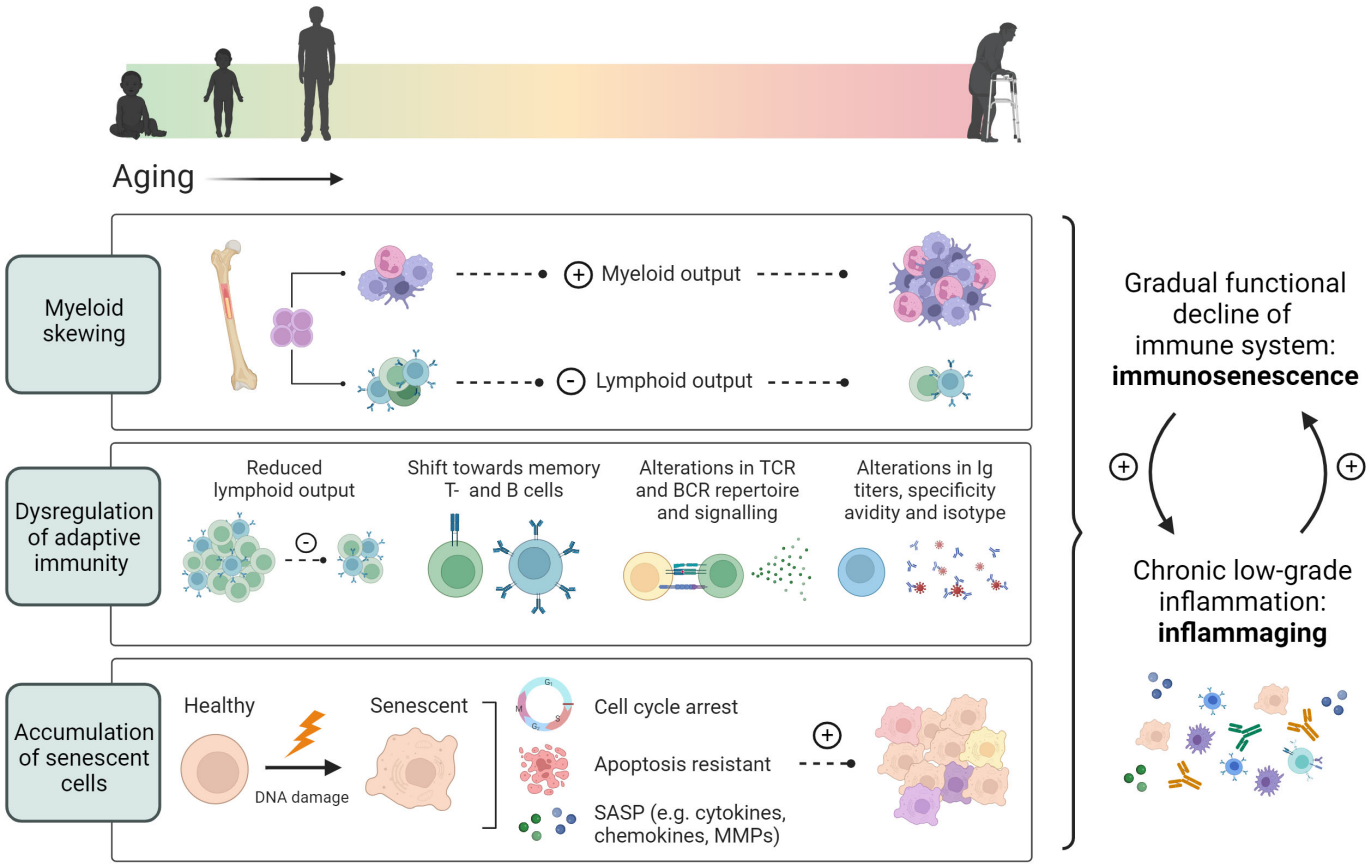
Age-associated immune alterations. Aging promotes skewing of hematopoietic stem cells towards myelopoiesis, resulting in a reduced lymphoid output from the bone marrow. In addition, thymic involution causes a decrease in the peripheral naïve T cell pool, while memory T- and B cells increase during aging. An age-associated reduction in TCR and BCR repertoire, and alterations in antibody secretion, specificity and avidity, further contribute to dysregulated adaptive immunity. Additionally, aging induces cellular senescence characterized by an induction of cell cycle arrest, apoptotic resistance and the production of a senescence associated secretory profile. These age-associated changes lead to an overall functional decline of the immune system termed immunosenescence and a chronic low-grade inflammation termed inflammaging.

Additionally, HSCs from aged (22-24 months) mice have been shown to down-regulate genes involved in lymphopoiesis while upregulating myeloid genes when compared to young mice (2-3 months), resulting in a skewing of hematopoiesis from lymphoid cells towards a myeloid output (75) (**Figure 1**). Hematopoietic myeloid skewing upon aging has furthermore been validated in the human hematopoietic system on a cellular level as well as through gene expression profiling (76). Notably, myelopoiesis can also be regulated through cholesterol efflux pathways and is promoted through hypercholesteremia (66, 67). Various studies have concluded both hypercholesteremia and hyperglycemia induced myelopoiesis to accelerate atherosclerosis and impair its resolution (67, 77, 78).

Aging additionally stimulates the clonal hematopoiesis of indeterminate potential (CHIP) which refers to the expansion of HSCs containing mutations unaffiliated to the development of hematopoietic malignancies (79). Notably, Jaiswal et al. have confirmed a 1.9-fold elevated risk of coronary artery disease in carriers of CHIP compared to non-carriers alongside an increase in coronary artery calcification and a 4-fold greater risk for early onset myocardial infarction (80). The risk of coronary artery disease positively associated with CHIP mutations of DNMT3A, ASXL1, JAK2 and TET2 as determined by whole genome sequencing.

Following the hematopoietic production of CLPs in the bone marrow, CLPs relocate to the thymus where they develop into competent and immunotolerant naïve T cells. However, the thymus already becomes fully developed in the early postnatal phase and shrinks with age, a process termed thymic involution. This deterioration of the thymus is associated with a gain of adipose tissue and severely affects the output of naïve T cells into the periphery, a prominent hallmark of immunosenescence (65, 81).

### 3.3 Accumulation of senescent cells

Immune cells can acquire a senescent phenotype upon aging as characterized by an irreversible loss of proliferative capacity, resistance to apoptotic cell death, and the acquisition of a senescence-associated secretory phenotype (82, 83) (**Figure 1**). This is partially due to the fact that most cells cannot divide indefinitely. With each replication cycle that occurs, telomeres shorten. When they become of insufficient length to support cell division, cells go into replicative senescence (64). In addition to classical replicative senescence induced through telomere shortening, driving factors working towards replicative senescence have been described as the accumulation of DNA damage and an overactive DNA damage response (82). The buildup of DNA damage with age is stimulated through the prolonged assault on the genetic code by stressors, resulting in genomic instability (63). The accumulation of DNA damage confers cytotoxicity, activating cell cycle arrest checkpoints, which serve as a signal to send cells down apoptotic pathways in order to protect them from damaged or misincorporated nucleotides becoming mutagenic (82). However, with prolonged cell cycle arrest, cells can become senescent, acquiring a resistance against apoptosis in the process (5, 84). The link between DNA damage and atherosclerosis has long been known, with coronary artery disease patients exhibiting significantly increased accumulation of DNA damage in peripheral blood lymphocytes, correlating with atherosclerosis severity and risk factors such as diabetes and hypercholesterolemia (85–87).

It must however be noted that the identification of senescent cells has proven a significant hurdle due to the lack of a single specific marker. The expression of cyclin-dependent kinase inhibitor p16^Ink4a^ and senescence-associated β-galactosidase (SA-β-gal) have for instance been extensively used in previous research as markers of cellular senescence (88–91). However, atherosclerotic plaques from human endarterectomy samples display an increase in p16^Ink4a^ concomitant to macrophage content (92). Additionally, a subclass of p16^Ink4a^ and SA-β-gal expressing macrophages has been observed as distinct from *bona fide* senescent cells induced through genotoxic stress *in vitro* (93). Expression of p16^Ink4a^ becomes upregulated upon monocyte differentiation while both SA-β-gal and p16^Ink4a^ exert control over macrophage polarization in response to immune stimuli (94, 95). p16^Ink4a^ deficiency in bone marrow derived macrophages additionally leads to a reduction in pro-inflammatory signaling (96). Therefore, it would not be advised to rely solely on SA-β-gal and p16^Ink4a^ as markers of senescence, especially in light of the prevalent role played by pro-inflammatory macrophages in plaque development. Similarly, expression of p53 and p21^cip1^ are often used as markers of senescence to address cell cycle arrest, proteins such as ataxia-telangiectasia mutated protein, ataxia telangiectasia and Rad3-related protein and phosphorylated histone H2AX are used as indicators of excessive DNA damage and an overactive DNA damage response, while the loss of the cell’s capacity to proliferate in its senescent state is attributed to reduced Ki-67 levels (82). However, on their own, none of these markers are specific for senescence. Because of this, it becomes paramount to apply them collectively to comprehensively investigate alterations in overarching systems typically dysregulated upon aging.

### 3.4 Pro-inflammatory senescence associated secretory profile

The SASP comprises of several pro-inflammatory factors, such as interleukins, chemokines, secreted proteases, and secreted extracellular matrix components (97). SASP acquisition by senescent cells is stimulated through nuclear factor kappa B (NFκB) signaling and the transcription factor CCAAT/enhancer-binding protein beta which is in turn epigenetically regulated by arginine methyltransferase 4 (98, 99). Vascular smooth muscle cells, endothelial cells and macrophages from human atherosclerotic plaques have been found to contain increased levels of activated NFκB (100, 101). The activation of NFκB can lead to the production of SASP factors such as Tumor necrosis factor α (TNFα), IL-1 β, IL-6 and IL-8, as well as various matrix metalloproteinases (MMP) (102). Eventually, the induction of both local and systemic chronic inflammation by the SASP can lead to tissue damage and fibrosis (97). An increased secretion of MMP-9 as well as a decrease in collagen production by senescent smooth muscle cells can furthermore lead to plaque instability through remodeling of the vessel wall (103, 104). Additionally, NFκB-mediated production of the chemokine monocyte chemoattractant protein-1 and the leukocyte receptors intercellular adhesion molecule 1 and vascular cell adhesion molecule 1 can subsequently drive the recruitment of monocytes (97). The SASP is also able to induce senescence in neighboring cells, exacerbating the accumulation of senescent cells with age (105). It has been speculated that the accumulation of senescent cells in the subendothelial space of atherosclerotic plaques contributes to the initial stages of atherosclerosis through the production of SASP markers such as IL-1α, TNFα and MMPs (91). However, in addition to the use of SA-β-gal and p16^Ink4a^ as the main markers of senescence in this study, it must be noted that non-senescent macrophages can similarly express IL-1α, TNFα and MMPs in a high capacity.

Thus, as we age, our immune system undergoes significant changes, including aging-induced alterations in hematopoiesis, accumulation of senescent cells, and the development of a pro-inflammatory senescence-associated secretory profile (SASP). These changes contribute to immunosenescence and can potentially increase CVD risk. Hematopoietic myeloid skewing and thymic involution impact immune cell production and functionality, while senescent cells exhibit an irreversible loss of proliferative capacity and contribute to chronic inflammation through adapting a SASP.

## 4 Aged adaptive immunity in atherosclerosis

### 4.1 T cell aging

The T cell compartment is strongly affected by aging. T cell numbers decrease as a consequence of reduced lymphoid output from the bone marrow and reduced generation of naïve T cells by the thymus (81). T cells additionally exhibit canonical features of cellular aging such as telomeric shortening, with CD4^+^ T cell telomere length decreasing by ~3000 bp between ages 20-60 years (7, 106). Accelerated telomere shortening has furthermore been observed in autoimmune disease, as well as in circulating lymphocytes of CVD patients (107–109). Simultaneously, as T cells age, their differentiation and secretory profile becomes skewed towards more extreme phenotypes evident from highly clonal regulatory and effector memory T cell compartments (15, 38). These factors can further contribute to alterations in the TCR repertoire observed in the T cell memory pool, impacting their capacity to elicit an immune response to a broad range of antigens upon aging (110). Age-associated TCR diversity is additionally affected by the persistent selection of T cells with a specificity against either antigens of viruses with lifelong retention (e.g. CMV and Epstein–Barr virus) or auto-antigens (81). Furthermore, this phenomenon can potentially serve as a driver of senescence as CMV seropositive octogenarians display a loss of CD27 and CD28 by CD4^+^ T cells, and a loss of CD45RO and CD27 in CD8^+^ T cells, as is characteristic for senescent T cells (69, 111, 112). Atherosclerotic plaques from patients who suffered a myocardial infarction as well as those with unstable angina similarly contain autoreactive CD4^+^ CD28^-^ T cells, while the expansion of CD4^+^ CD28^-^ T cells has been attributed to an increased cytotoxic affinity towards endothelial cells in acute coronary syndromes (113–116). Moreover, CD4^+^ CD28^-^ T cells in coronary artery disease express increased levels of granzymes (Gzm) A and B, perforin, granulysin and IFNγ upon *ex vivo* stimulation, indicative of a highly cytotoxic and inflammatory phenotype (117). In addition to expansion of CD4^+^ CD28^-^ T cells, CD8^+^ T cells with high expression of CD57 have been observed by Grievink et al. in both elderly healthy populations and coronary artery disease patients as compared to the young healthy population (118). Increased expression of senescent markers CD57 and killer cell lectin-like receptor subfamily G member 1 (KLRG1) drive terminal differentiation and have also been found to occur in terminally differentiated effector T memory cells re-expressing CD45RA (TEMRA) in the peripheral blood of CVD patients (38). TEMRA cells express cytotoxic and inflammatory genes driven by p38 MAPK signaling as well as a senescent phenotype characterized by impaired proliferation and mitochondrial dysfunction (119, 120). Furthermore, the association of cell membrane bound CD153 (CD30L) with the TCR/CD3 on senescent T cells has been found to stimulate T cell signaling and activation, while CD153^-/-^ mice exhibit a reduction in aging-associated T- and B cells (121). The blocking of CD153 in atherosclerotic Ldlr^-/-^ mice using monoclonal antibodies has additionally been shown to lead to a reduction in lesion size and adventitial T cells (122). Interestingly, aging also results in accumulation of cholesterol in the T cell membrane (123). This may promote cellular senescence, as T cells from middle-aged Ldlr^-/-^ mice with a specific deficiency in cholesterol efflux transporters ABCA1 and ABCG1, showed upregulated Cyclin dependent kinase inhibitor 1a (Cdkn1a) and impaired proliferative capacity (124). At the same time, accumulation of cholesterol in T cells from T cell-specific Abca1/Abcg1 deficient middle-aged mice increased apoptosis, contributing to a reduction in the peripheral T cell pool and atherosclerotic plaque development.

The shift towards more extreme T cell phenotypes upon aging becomes evident in the immune compartments of aged atherosclerotic Ldlr^-/-^ mice which contain increased pro-atherogenic T cells producing IFNγ as well as expanded regulatory and effector T cell phenotypes (15). Additionally, an age-associated increase in pro-inflammatory CD8^+^ GzmK^+^ T cells was observed within the atherosclerotic aorta of Ldlr^-/-^ mice, as well as in atherosclerotic plaques from CVD patients (38, 125). Atherosclerotic murine CD8^+^ GzmK^+^ T cells primarily displayed an effector and memory phenotype by increased expression of Ccl5, Nkg7, Cd52, and Id2 whilst the expression of exhaustion markers Tox-1, Eomes and Lag3 was more prevalent in the CD8^+^ GzmK^+^ T cell population from aged Ldlr^-/-^ mice compared to young atherosclerotic mice (15). Extracellular GzmK has furthermore been shown to exacerbate secretion of aging related inflammatory factors such as IL-6, chemokine C-C motif ligand (CCL) 2, and chemokine CXCL1 in fibroblasts both in the presence of IFNγ and without (126). Moreover, in human carotid artery plaques, GzmK expressing CD8^+^ T cells were shown to exhibit plaque specific clonal expansion and displayed high levels of genes involved in T cell activation such as CD69, FOS and FOSB (38). Nevertheless, whether activation and clonal expansion of CD8^+^ GzmK^+^ T cells in human atherosclerotic plaques is achieved through stimulation of TCRs specific against known atherosclerosis associated auto-antigens such as oxLDL, ApoB100 or HSP60/65, and whether CD8^+^ GzmK^+^ are causally associated with atherosclerotic CVD remains to be investigated.

Additionally, deterioration of CD8^+^ T cell tolerance has furthermore been suggested in aged ApoE^-/-^ mice (78–85 weeks) on a standard chow diet due to decreased expression of Ccr7, S1pr1 and Sell, involved in regulating lymphocyte egress from SLOs, as well as an upregulation of genes involved in cytotoxic activity and cytokine production such as Slamf7, S100a6, Gzmk, Nkg7, Ccl5, Ifng and Prf1, and a dysregulation of exhaustion associated genes such as Pdcd1, Tigit, Lag3 and Havcr2 (127). Integration of the mouse data with single-cell RNA sequencing (scRNAseq) data from human coronary and carotid plaques showed similar transcription profiles between species concerning pathways involved in the egress from lymphoid organs and activation of CD8^+^ T cells.

Aging not only induces the expansion of pro-inflammatory and cytotoxic T cell subsets, but also stimulates an increase in T cells with regulatory phenotypes. An overall increase of Tregs was observed in the atherosclerotic aorta of aged Ldlr^-/-^ mice alongside a heightened expression of functional Treg markers such as Ctla4, Lag3, and Tnfrsf4 (OX40) and genes encoding for the IL-35 cytokine as compared to young Ldlr^-/-^ mice (15). Similar upregulation of genes indicative of Treg activity was demonstrated in plaques from human endarterectomy specimens as compared to patient PBMCs (38). Moreover, Tregs show clonal expansion in the human carotid plaque. Previously, it has been reported that Treg functionality can decrease upon aging (128, 129). Whether aging also impacts the immunosuppressive capacity of Tregs in the atherosclerotic environment, remains to be elucidated. It may be that conversion from Tregs towards exTregs as described before, can significantly alter their function, for instance causing the acquisition of a cytotoxic phenotype. But while some studies describe such a maladaptive Treg conversion (28, 29), others showed that Tregs in human plaques did not show any co-expression of FOXP3 and RORC nor showed any evidence of Treg-Th17 conversion by complementary RNA velocity analysis (38), warranting further research into the function of Tregs in the atherosclerotic microenvironment.

### 4.2 B cell aging

Aging is associated with significant changes in the B cell compartment including changes in B cell subsets and the emergence of age-associated B cells (ABCs), as well as alterations in the BCR repertoire and antibody responses. In addition to an aging-associated decrease in self renewal of HSCs in the bone marrow, a reduction in the differentiation of progenitor B cells, precursor B cells and immature B cells has for instance been correlated to the presence of aged bone marrow stroma in aged mouse models (73, 130). This impaired differentiation can be partially attributed to a reduced capacity of the murine progenitor B cells to respond to stromal derived IL-7 on which they depend for their survival (131, 132). Similar to T cells, a reduction in B cell precursors in lymphoid organs could conceivably impact the diversity of the BCR repertoire. However, research concerning a potential age-related deterioration of BCR repertoire diversity in humans has given rise to conflicting results. Although a reduced BCR repertoire has been demonstrated in the peripheral blood and lymph nodes of the elderly population, an increase in BCR diversity has for example been observed in aged spleens obtained from archived human biopsy samples (133–135). In mice, it was recently shown that the BCR repertoire diversity decreases upon aging. Interestingly, dietary restriction has been shown to attenuate the observed aging-associated decrease in BCR diversity in C3B6F1 mice, achieving a similar retention of BCR repertoire in mice started on a restrictive diet during mid- life as compared to mice on a chronic diet (136, 137).

The effect of aging on B cell subsets has been shown to extend to B2 cells by decreased levels of MZ B cells in aged BALB/c mice (138). MZ B cells originating from aged C57BL/6J mice additionally display an impaired ability to generate antibodies in response to T cell independent activation by trinitrophenyl-lipopolysaccharide (139). Nevertheless, aging induced changes in MZ B cell frequency and function are poorly understood in the context of atherosclerosis. However, an age-associated decrease in the plasma levels of B cell activating factor (BAFF), which is essential for the survival of both FO- and MZ B2 cells, but not peritoneal B1 cells, has been observed in healthy individuals (140–142). In models of experimental atherosclerosis, B2 cell depletion achieved through the antibody-mediated blocking of the BAFF receptor has resulted in decreased B cell zones in the spleen of hyperlipidemic ApoE^-/-^ mice while limiting the progression of atherosclerotic plaques (143). However, neutralization of BAFF itself has unexpectedly led to the promotion of atherosclerotic plaque progression even though both FO- and MZ B2 cells were similarly depleted (144). This study suggests BAFF to convey atheroprotection through the binding of transmembrane activator and CAML interactor (TACI), inhibiting the production of proatherogenic CXCL10 by IRF7-dependent toll-like receptor (TLR) 9 signaling. Aging furthermore impedes on class switch recombination and somatic mutation in B2 cells through a reduction in the expression of E47 transcription factor and activation-induced cytidine deaminase as well as through a decrease in their DNA binding capacity (145). Nevertheless, whether these aging associated reductions are specific to a particular subset of B2 cells is unclear as this study magnetically sorted murine splenic B cells using anti-B220 and did not differentiate between FO- and MZ B2 cells.

Multiple studies have demonstrated the atheroprotective effects of B1 cells through the secretion of IgM targeting OSEs (143, 146). Additionally, the levels of B1 cells and IgM specific for either ApoB100 or various OSEs, have been shown to negatively correlate with CVD risk (147–151). However, in the peripheral blood of the elderly population an overall decrease in the frequency of B1 cells can be observed as well as decreased levels of circulating atheroprotective oxLDL-specific IgM compared to young individuals, which shows an additional reduction in age-matched coronary artery disease patients (118, 152). Whether aging also impacts the efficacy of oxLDL-specific IgM antibodies is not yet known. The aging-associated reduction of B1 cell frequency in CVD patients has been correlated to the inhibition of XBP-1 and Blimp-1 transcription factors and an increased presence of Pax5, a marker expressed by most B cells but which is lost upon plasma cell differentiation (152). Furthermore, the expression of C-X-C chemokine receptor type (CXCR) 4 by B1 cells has been demonstrated to correlate to levels of OSE specific IgM antibodies in a cohort of CVD patients (151). This correlation has mechanistically been demonstrated in aged ApoE^−/−^ mice by CXCR4 dependent production of IgM by B1a cells (151). However, a comparison of B1a and B1b cells through adoptive transfer experiments in aged ApoE^-/-^ Rag1^-/-^ mice has shown B1b cells to be the most efficient producers of OSE specific IgM (153). B1b cells additionally migrate to the spleen more efficiently than B1a cells through increased expression of C-C chemokine receptor type (CCR) 6.

Expansion of a late memory IgD^-^ CD27^-^ double negative (DN) B cells has furthermore been described in the peripheral blood of the elderly population as well as in autoimmune diseases (154–156) but their role in atherosclerosis is poorly understood. DN B cells display senescent features indicated by telomeric shortening, a reduced proliferative capacity and the expression of SASP markers such as IL-6, TNFα, p16^INK4^ and pro-inflammatory micro-RNAs (154, 157, 158). Late memory DN B cells are a heterogenous population which contain CD21^low^ CD11c expressing age-associated B cells (ABCs) (159, 160). ABCs can produce high levels of autoantibodies and pro-inflammatory cytokines, such as IFNγ, TNFα, IL-6 and IL-1β, following activation via TLR7 and TLR9 (161). Similar to DN B cells, ABCs accumulate during aging and in autoimmune diseases, such as rheumatoid arthritis and multiple sclerosis (159, 162–164). Aging-induced formation of fat-associated lymphoid clusters likewise display an accumulation of ABCs in a manner dependent on the NOD-, LRR- and pyrin domain-containing protein 3 inflammasome (165). This finding could prove relevant for atherosclerotic pathogenesis due to the presence of perivascular adipose tissue involved in the local stimulation of plaque development (166). In light of this, the presence of ABCs has recently been uncovered in atherosclerotic aortas of aged Ldlr^-/-^ mice using scRNAseq and flow cytometry and in carotid plaques obtained from CVD patients (15). In comparison to other B cell clusters observed in the aortic arches of atherosclerotic mice, ABCs displayed a distinctive expression profile characterized by upregulated Tbx21, Itgam, Itgax (encoding CD11c), Fas, Zbtb20, and Ighg3. The expansion of ABCs was additionally shown to be more prominent in aortic arches of Ldlr^-/-^ and ApoE^-/-^ mice as compared to healthy age-matched C57BL/6 mice. In line with these research findings, Pattarabanjird et al. describe an age-associated increase of CD11c expressing B cells, containing ABCs, in the spleen and bone marrow of ApoE^-/-^ mice concomitant with an increase in aortic plaque size (167). Furthermore, ABCs have been demonstrated as highly potent antigen presenting cells *in vivo* through transcriptome analysis of aorta-enriched ABCs from Ldlr^-/-^ mice (15), as well as *in vitro* by antigen-specific T cell proliferation assays and through the use of multiphoton microscopy in which ABCs display an increased stability of antigen specific B-T cell interactions (15, 168). There have been indications of ABCs being able to skew T cell differentiation towards a Th17 phenotype through their antigen presenting capability, but whether this also occurs in atherosclerosis context remains to be investigated (15, 169). Interestingly, the frequency of CD11c expressing B cells positively correlated to plaque severity in cardiac catheterization patients assessed through coronary angiography (167).

**Figure 2:**
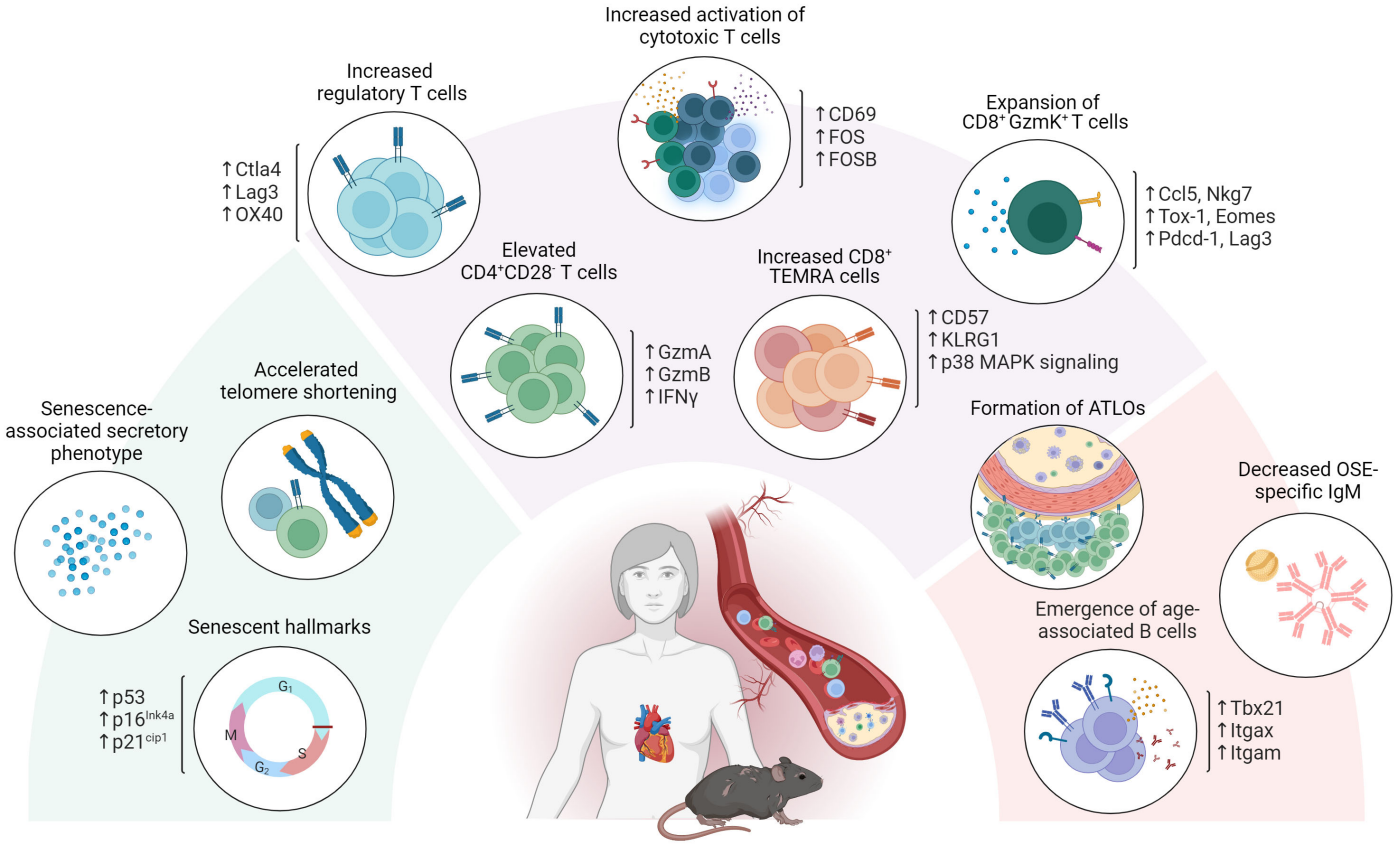
Age-associated adaptive immunity in atherosclerosis. Aging severely affects the role of adaptive immunity in atherosclerosis development. In addition to exhibiting senescent hallmarks such as telomeric shortening, cell cycle arrest and adopting a senescence associated secretory profile, atherosclerosis specific T- and B cell dynamics are affected through the emergence of maladaptive age-associated subsets including CD4^+^ CD28^-^ T cells, CD8^+^ GzmK^+^ T cells and age-associated B cells (ABCs). Atherosclerosis specific humoral immunity additionally shows a decrease in OSE specific IgM and an increase in BAFF, essential for B2 cell survival upon aging.

Overall, aging severely affects the role played by adaptive immunity in atherosclerosis development. In addition to an increase in circulatory pro-inflammatory cytokines and reduced OSE specific IgM, this has become particularly evident from the emergence of maladaptive subsets in both the T- and B cell compartments (i.e. CD4^+^ CD28^-^ T cells, CD8^+^ GzmK^+^ T cells and ABCs) as observed upon aging as well as in CVD (**Figure 2**).

### 4.3 T-B cell interactions and deteriorating secondary lymphoid tissues with age

Aging stimulates early Tfh differentiation in mice as evident from increased numbers of pre-cursor Tfh cells in draining lymph nodes through modulating expression of RBPJ, the Notch-associated transcription factor (170). Additionally, an increase in activated memory Tfh cells has been observed in aged atherosclerotic mice compared to their younger counterparts (34). The formation of spontaneous germinal centers and autoantibody production has furthermore been reported in relation to CD30L expressing Tfh cells with high expression of the SASP factor osteopontin and senescence-associated heterochromatic foci in the nuclei, contributing to autoimmunity in lupus mouse models (171–173). Similar overactivation of the Tfh–GC B cell axis has been previously shown to exacerbate atherogenesis in ApoE^-/-^ mice deficient for Qa-1–restricted CD8^+^ regulatory T cells, a phenotype subsequently rescued by antibody treatment specific against inducible costimulator-ligand (ICOSL) to inhibit Tfh activity (34). In contrast, a reduction in Tfh expansion following influenza vaccination has been observed in the elderly population as compared to individuals aged 18-36 years (174). In line with this finding, Silva-Cayetano et al. have shown that following immunization with NP-KLH, a model antigen to assess vaccine responses, an aging-associated decrease in germinal center formation and quality of antibody responses was observed through spatial dysregulation of Tfh cells in BALB/c mice (175). Aged Tfh cells overexpress CXCR4, impeding on their colocalization with FDCs necessary for their priming. This impairs effective Tfh-B cell interactions and Tfh support of B cell survival, proliferation and differentiation towards GC B cells. Notably, disruptions in T-B cell interactions impact Tfh cell proliferation as well, as evident by the accumulation of Tfh cells in the spleens of irradiated Ldlr^-/-^ mice reconstituted with MZ B cell deficient bone marrow (57). This study showed that MZ B cells exert control over the Tfh response to a high cholesterol diet in a PD-L1 and ATF3-dependent manner. In absence of MZB cells, atherosclerosis was aggravated. In line with this, it has been shown that inhibition of the Tfh-GC B cell axis through PD-L1 expressing B cells protects against atherosclerosis (52).

In addition to an altered function of aged Tfh cells, SLOs display age-related deterioration, decreasing in both size and complexity of their architecture (176). Firstly, the trafficking of T cells through the SLO scaffolding becomes impeded by an aging induced reduction in fibroblastic reticular cells which lay down the extracellular matrix (177, 178). Additionally, this reduction affects the deposition of IL-7 on the extracellular matrix which is essential for the preservation of naïve T cells (179). Similarly, SLOs experience a decrease in the area of the light zone alongside the therein contained FDCs necessary for priming Tfh cells (175, 180). Overall, these age-related changes impede the correct function and localization of cells from the adaptive immune system and can lead to aberrant germinal center responses. Nevertheless, how the aging induced deterioration of SLO architecture and function might impact either pathogenic or protective humoral responses relating to atherosclerotic CVD risk is still understudied. This is especially true as the development of artery tertiary lymphoid organs (ATLOs) have been reported in aged atherosclerotic ApoE^-/-^ mice (181, 182). Though ATLOs occur at sites of chronic inflammation, they include dedicated T cell zones, B cell follicles and germinal centers and are capable of regulating B cell responses in a manner similar to that of SLOs (183). However, ATLOs exhibit significant angiogenesis and high endothelial venules which facilitate the recruitment of naïve T- and B cells to the lymphoid tissue (182, 184). As such, ATLOs have been suggested to be involved the pathogenesis of advanced atherosclerosis by stimulation of primary immune responses (183).

## 5 Immunoaging as biomarker or therapeutic target to combat atherosclerotic CVD

Currently few biomarkers exist that can indicate risk of major adverse cardiovascular events (MACE). Increased plasma levels of low-density lipoprotein cholesterol (LDL-C), non–high-density lipoprotein cholesterol (non-HDL-C), IL-18, and TNFα, IL-6 and high-sensitivity C-reactive protein (hsCRP) have previously been associated with poor cardiovascular outcomes (185–187). However, inflammatory risk assessed by circulating hsCRP is for example unlikely to represent the full extent of atherosclerosis associated inflammation, as hsCRP is solely driven by circulating IL-6 levels (24). With an aging population at increased risk for CVD and cardiovascular related death, it is of the utmost importance to discover new biomarkers for assessing atherosclerotic disease state to inform timely and appropriate clinical intervention. As increased incidence of atherosclerotic MACE occurs in parallel to aging, the breakdown of immunotolerance and increased susceptibility for the development of autoimmune responses in the elderly population could provide valuable biomarkers for this exact purpose (127, 188–190). As such, the systemic expansion of atherogenic age-associated immune cells and their secreted products (e.g. autoantibodies), could be explored to stratify individuals at risk for atherosclerosis driven MACE (118, 154, 191). Illustratively, the trajectory of immune aging by the algorithmic summary of elements of immunosenescence, termed the IMM-AGE score, has been found to predict all-cause mortality more accurately than an individual’s chronological age and established risk factors (192). Additionally, an increased IMM-AGE score was observed for patients diagnosed with cardiovascular disease in the Framingham Heart Study. Nevertheless, the predictive clinical value of the IMM-AGE score for risk of MACE has not yet been determined in a prospective cohort.

The current treatment of CVD relies on risk factor management, whilst no treatments directly attacking inflammatory processes in the vessel wall are currently available. The necessity for such treatments is illustrated by the persistence of cardiovascular events as well as residual inflammatory risk in patients recruited in contemporary lipid lowering trials (193–195). Current strategies therefore additionally focus on targeting the underlying immunity involved in CVD pathogenesis. The blocking of IL-1β with Canakinumab (CANTOS trial) as well as the Low-Dose Colchicine-2 (LoDoCo2) trial have illustrated the effectiveness of modulating inflammation for the prevention of atherosclerosis induced CVD events, independent of lipid levels (196, 197). However, the increased rate of fatal sepsis in these trials highlights the need for atherosclerosis specific immune modulation in contrast to a general systemic reduction in inflammation. As such, the safety and biological efficacy of Treg expansion by low dose IL-2 in patients with ischemic heart disease was confirmed in the LILACS study, while its application for the reduction of vascular inflammation is currently under investigation in the IVORY trial (198, 199). Similarly, reduction of mature B cells by Rituximab, a monoclonal antibody against CD20, in the RITA-MI study did not report any drug related safety issues (200). Administration of anti-CD20 in mice has been shown to deplete pro-atherogenic B2 cells as opposed to B1 cells, attenuating atherosclerosis (201, 202).

With an increased life expectancy, the aging population similarly increases, which additionally emphasizes the need for strategies to delay or reverse age-associated immunosenescence. Significant attention goes towards clearance of senescent cells by a class of drugs called senolytics. In atherosclerotic mice, treatment with either Quercetin or Navitoclax which target anti-apoptotic Bcl-2 family proteins, resulted in decreased atherosclerotic lesion size and circulating pro-inflammatory factors (91, 203–205). However, through the targeting of apoptotic pathways, Quercetin and Navitoclax can non-specifically affect healthy cells as well, resulting in severe thrombopenia and neutropenia (206–208). Though the use of proteolysis-targeting chimera technology has been shown to ameliorate the platelet toxicity of Bcl-2 inhibitors, and next generation senolytics with higher selectivity are emerging, the use of senolytics for the clinical treatment of CVD still seems a ways off (209). As such, specific depletion of maladaptive age-associated immune cell subsets warrants further investigation. However, a first step in guiding this process would be high dimensional characterization of aged immunity in atherosclerosis in order to uncover more specific markers for targeting through therapeutic intervention, an effort currently led by single cell technologies.

## 6 The external validity of immunological findings from mice studies

Age matching laboratory mice to the actual atherosclerotic CVD patient population is invaluable for improving the external validity of research concerning the immune components at play in atherosclerotic plaque progression. However, laboratory mice are not without their limitations. Laboratory mice are kept within highly standardized environments in which the temperature, humidity, light cycles and diets (among others) are regulated. This is especially true in immunological research in which specific pathogen-free (SPF) mice have become the standard. SPF husbandry impacts the gut microbiome heterogeneity and maturation of the murine immune system, limiting its translatability to human immunity matured through exposure to both acute and chronic pathogens (210, 211). A noteworthy development to circumvent an immature immune system caused by limited exposure to pathogens can be found in “wildling” mice born through the transfer of lab-strain embryos into wild mice (212). The immune landscape of wildling mice closely resembles that of wild mice. Additionally, for both human clinical trials targeting the adaptive immune system through administration of a CD28-superagonist (CD28SA) or the innate immune system by anti-TNF-α treatment in sepsis, Rosshart et al. have found C57BL/6 wildling mice to be better predictors of clinical outcome as compared to conventional laboratory mice (212). However, whether research into the inflammatory aspects of atherosclerotic pathogenesis could benefit from using wildling mice on an atherosclerosis prone background to increase their external validity is currently unclear. Moreover, intensifying research efforts into immunosenescence in CVD patients may contribute to the identification of biomarkers for the stratification of patients as well as the identification of therapeutic targets specific to the aged immune landscape at play in CVD in order to mitigate disease burden.

## 7 Conclusion and future perspectives

Given the demographic shift towards an elderly population, it has become paramount to elucidate the role of age-associated maladaptive immunity in the progression of atherosclerotic CVD occurring in parallel to aging. Current advances in single cell technology have shed light on the age-associated dysregulation of atherosclerosis specific T-B cell dynamics leading to the expansion of atherogenic, pro-inflammatory and cytotoxic cellular subsets. In the future, this information could contribute to the identification and therapeutic depletion of maladaptive immunity to reduce atherosclerotic CVD risk in the elderly population. However, the in-depth characterization of age-associated maladaptive immunity involved in atherosclerotic CVD pathogenesis, as well as the age-specific immune cell organization across the plaque environment and lymphoid tissues are an ongoing process. Both these factors will need to be explored further to improve upon the classical stratification of the atherosclerotic CVD patient population seeking treatment at advanced age.

## Author contributions

RPMS: Conceptualization, Visualization, Writing – original draft. ACF: Conceptualization, Supervision, Visualization, Writing – review & editing, Funding acquisition.

## Glossary

**Table:**

ABC	age-associated B cells
apoB100	apolipoprotein B
ApoE	apolipoprotein E
APRIL	a proliferation-inducing ligand
ATLO	artery tertiary lymphoid organ
BAFF	B cell activating factor
BAFFR	B cell activating factor receptor
BCR	B cell receptors
BTLA	B- and T-lymphocyte attenuator
CCL	chemokine C-C motif ligand
CCR	C-C chemokine receptor type
CHIP	clonal hematopoiesis of indeterminate potential
CLP	common lymphoid progenitor cells
CMV	cytomegalovirus
CVD	cardiovascular disease
CXCL	C-X-C Motif Chemokine Ligand
CXCR	C-X-C chemokine receptor type
DC	dendritic cell
DN B cells	IgD-CD27- double negative B cells
FDC	follicular dendritic cell
FO B cells	follicular B cells
FOXP3	forkhead box P3
GC B cell	germinal center B cell
Gzm	Granzyme
HSC	hematopoietic stem cell
hsCRP	high-sensitivity C-reactive protein
HSP	heat shock protein
ICOSL	inducible costimulator-ligand
IFNγ	Interferon gamma
Ig	immunoglobulin
IL	interleukin
KLRG1	killer cell lectin-like receptor subfamily G member 1
LDL	low density lipoproteins
LDL-C	low-density lipoprotein cholesterol
Ldlr	low density lipoprotein receptor
MACE	major adverse cardiovascular events
MHC	major histocompatibility complex
MMP	matrix metalloproteinases
MZ B cell	marginal zone B cells
NFκB	nuclear factor kappa B
non-HDL-C	non–high-density lipoprotein cholesterol
OSE	oxidation specific epitopes
oxLDL	oxidized low-density lipoprotein
SASP	senescence associated secretory profile
SA-β-gal	senescence-associated β-galactosidase
scRNAseq	single-cell RNA sequencing
SLO	secondary lymphoid organ
SPF	specific pathogen free
TACI	transmembrane activator and CAML interactor
TCR	T cell receptor
TEMRA	effector T memory cells re-expressing CD45RA
Tfh	T follicular helper
Tfr	follicular regulatory T helper
Th	T helper
TLR	toll-like receptor
Tregs	regulatory T cells
